# A Multi‐Axis Plantar Load Measurement Platform for the Assessment of Foot Disorders: Comparison of Patients With Psoriatic Arthritis to a Healthy Control Cohort

**DOI:** 10.1002/jfa2.70216

**Published:** 2026-10-14

**Authors:** Dominic Jones, Lefan Wang, Max Houghton, Jennifer Jones, Richard A. Wilkins, Heidi J. Siddle, Graham J. Chapman, Ali Alazmani, Peter Culmer, Philip S. Helliwell

**Affiliations:** ^1^ STORM Lab UK School of Electronic and Electrical Engineering University of Leeds Leeds UK; ^2^ Institute for Manufacturing University of Cambridge Cambridge UK; ^3^ Institute of Design, Robotics and Manufacturing School of Mechanical Engineering University of Leeds Leeds UK; ^4^ Leeds Institute of Rheumatic & Musculoskeletal Medicine, University of Leeds Chapel Allerton Hospital Leeds UK; ^5^ Podiatry Department Leeds Teaching Hospitals NHS Trust Leeds UK; ^6^ NIHR Leeds Biomedical Research Centre Leeds Teaching Hospitals NHS Trust Leeds UK; ^7^ School of Health, Social Work and Sport University of Lancashire Preston UK; ^8^ Rheumatology Department Bradford Teaching Hospitals NHS Foundation Trust St Luke's Hospital Bradford UK

**Keywords:** gait, medical instrumentation, plantar force measurement, plantar shear measurement, psoriatic arthritis

## Abstract

**Background:**

Accurate assessment of plantar loading, including shear forces, is crucial for understanding foot pathologies such as dactylitis in psoriatic arthritis (PsA). Existing devices predominantly measure plantar pressures, neglecting important shear stresses which may initiate dactylitis. This study presents a novel multi‐axis plantar load measurement platform to address this gap.

**Methods:**

The study utilised a newly developed plantar load measurement system, comprising a Hall‐effect‐based magnetic sensing array, custom data logging electronics, and tailored data processing software. The system's efficacy was evaluated in a pilot feasibility study involving patients with PsA with no, current, or previous dactylitis, and healthy controls. Participants walked barefoot on the platform, capturing plantar shear and pressure data.

**Findings:**

Shear stresses were notably higher in the second to fourth rays compared to other regions, particularly in the left foot, aligning with the hypothesis that repetitive local trauma might initiate dactylitis in PsA. However, no consistent differences in stresses were observed between diagnostic groups, likely due to small sample sizes and gait alterations associated with pain. A marked asymmetry was observed between the right and left feet, suggesting dominant‐foot placement effects.

**Interpretation:**

The new platform demonstrates potential clinical utility in capturing comprehensive foot‐loading profiles, including critical shear stresses previously under‐assessed. Although the current pilot study was limited in number, the results support the theory that local mechanical stresses may contribute to PsA‐related dactylitis. Future larger‐scale studies are needed to clarify these findings and overcome limitations related to altered gait patterns due to foot pain.

## 1 Introduction

Electronic foot‐load measurement systems measure the load distribution transmitted through the plantar surface of the foot. Distinct from force platforms which measure single‐point ground‐reactions, these systems record the spatial pattern and magnitude of the contact load. These data can thus provide an insight into how the foot and ankle distribute body weight through the plantar surface of the foot during static or dynamic activities [1]. Consequently, foot‐load measurement systems are used across a variety of fields including biomechanics research (e.g., investigations into human gait), commercial product development (design of supportive footwear), sport (biometrics) and healthcare [2, 3].

A range of foot‐load instrumentation has been developed to measure *plantar loading* in healthcare. Systems fall into two categories: wearable ‘in‐shoe’ systems placed within footwear or static platforms that are stepped on directly. These are complementary in function; in‐shoe systems capture information on the plantar loading that occurs while wearing footwear, foot platforms directly measure the unshod foot, which can highlight abnormal load profiles or features that would be masked or attenuated by footwear. A recent review considers in‐shoe systems in detail [4] but the focus concerns foot‐load platforms which have progressed from early research [5] to become commercially available (e.g., emed, novel GmbH). While some aspects of their form and measurement function vary, current commercial offerings are limited to the measurement of plantar *pressure*. This is an important aspect, as pressure alone neglects the shear stresses which also occur at the plantar surface. Shear stress forms an important part of plantar load; plantar pressure is the vertical component of load during gait and shear stress, acting parallel to the foot, forms the tangential component of plantar load. In a systematic review and meta‐analysis of plantar shear stress in the diabetic foot, shear has been associated with progression of disease [6]. Furthermore, patients with diabetes and a history of ulceration exhibit greater shear stress than their ulcer‐free counterparts and those without diabetes. The importance of shear stresses has been further demonstrated following the development of a novel system, the STrain Analysis and Mapping of the Plantar Surface (STAMPS) system, an innovative system using a plastically deformable insole with a stochastic speckle pattern, developed to measure peak plantar shear strain (SMAG) in people with diabetes [7]. Participants with a recently healed diabetic foot ulcer exhibited elevated shear strain characteristics compared with a low‐risk group [8]. In other diseases, such as psoriatic arthritis (PsA), vertical plantar load (peak plantar pressure) at the 2nd and 4th toes and 2nd and 4th MTP joints was not different in those with and without dactylitis [9], but further information is needed on the shear stresses in this condition.

Acknowledging the importance of plantar shear, a range of researches have investigated the development of instrumentation capable of measuring one or more plantar shear‐load components, detailed in a recent comprehensive review [10]. To date, the seminal research by Yavuz et al. [11] represents one of the few significant advancements, reporting a platform capable of multi‐axis load measurements. However, Yavuz's platform is limited by a small device size and the presence of partial contact within the force sensing regime. Motivated by these limitations, we developed a platform to measure discrete point loading of 3‐D forces, while patterning a larger sensing surface than previously observed, aiming to provide a more practical and effective tool for detailed plantar load analysis, particularly in a clinical setting.

A hallmark clinical feature of psoriatic arthritis (PsA) is dactylitis, or inflammation of an entire digit. In dactylitis, inflammation occurs in several tissues of the digit, including the joints, the tendons, the bones, the entheses and the soft‐tissues [12]. Dactylitis occurs in about 40% of patients with PsA and is a severity marker for the disease. It occurs in the foot more commonly than the hand, and in the second and fourth toes in particular [13]. It has been hypothesised that dactylitis results from repeated trauma, which may initiate inflammation, the so‐called ‘deep’ Koebner phenomenon [14]. The load bearing function of toes during activities of daily living supports the mechanical pathogenesis hypothesis of toe dactylitis, but few biomechanical studies have been conducted examining this relationship. A previous study from this group failed to demonstrate any differences in plantar pressure profiles between patients with dactylitis and those without dactylitis [15]. In a pilot study using newly developed technology, we examine the magnitude of shear stresses in the forefoot of patients with and without dactylitis, and in normal controls. We hypothesised that patients with PsA and active/previous toe dactylitis will experience higher shear stress under the forefoot/toes compared with PsA patients without dactylitis and healthy controls.

## 2 Methods

### 2.1 Development of the Platform

The system design was heavily informed through clinical consultation to form a set of technical requirements for a walkway‐integrated sensing system. These key aspects are presented in Figure 1. The system comprises of three main components: (1) the sensing plate, (2) the custom datalogging system, and (3) the contact analysis software.

**FIGURE 1:**
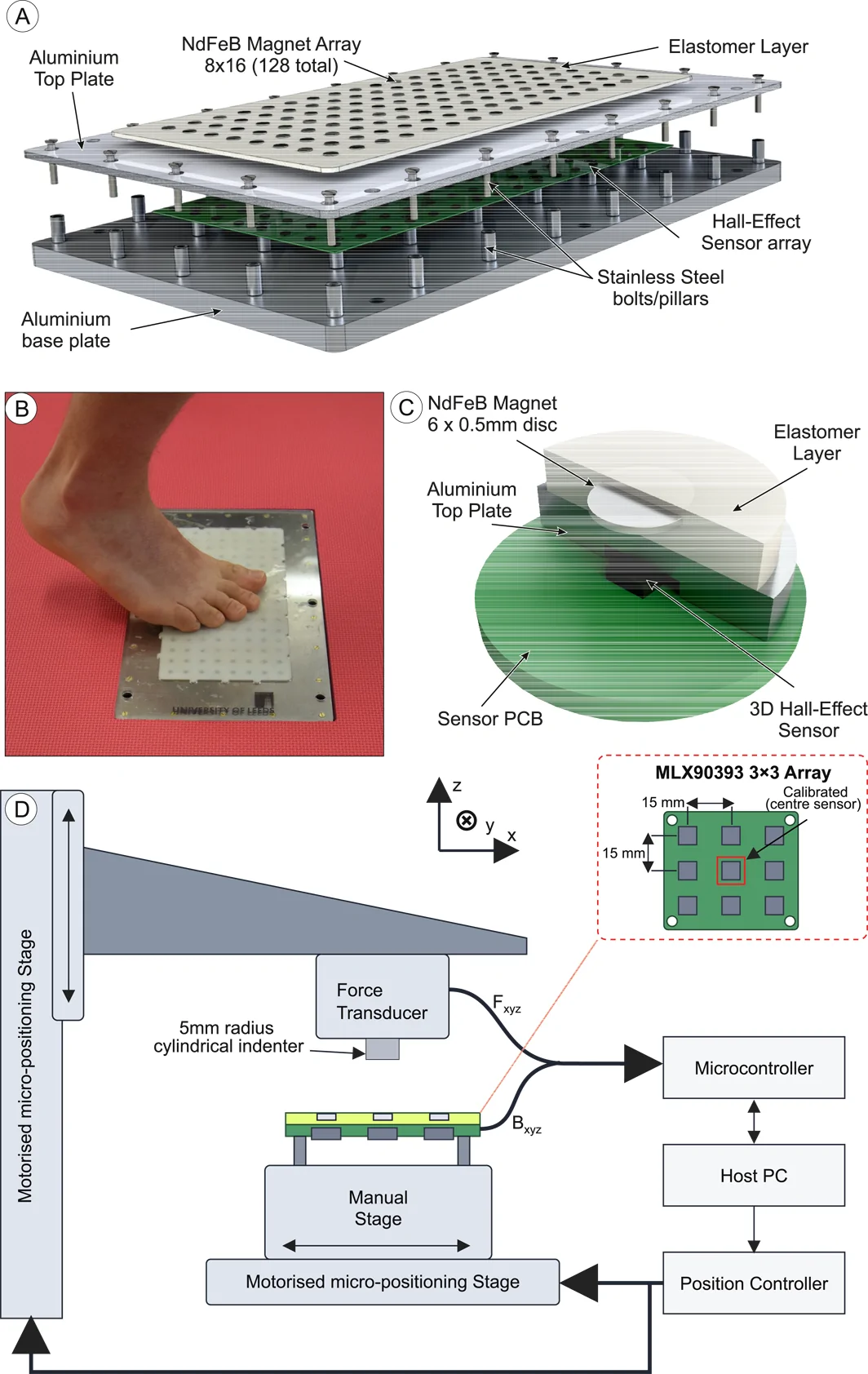
Schematic representation of the essential components of the measurement system (A) an exploded view of the layers comprising the system, showing the active sensing area of 8 × 16 sensors (covering an area of 120 × 240 mm), (B) the system in use during data capture, (C) the composition of an individual sensing node and (D) the apparatus used for two‐stage calibration of the sensor nodes.

The system adapts the Hall‐Effect‐based tactile sensing technologies previously developed in our previous work [16, 17]. In total, 128 three‐axis tactile sensors were integrated into the plate to form the sensing array. Each individual sensing unit is hereafter referred to as a ‘node’. Each node is comprised of three main components: a three‐axis Hall‐Effect sensor (MLX90393, Melexis), a layer of elastomer, and an embedded neodymium magnet.

#### 2.1.1 Sensor Design and Calibration

The sensor operates by measuring the varying three‐axis magnetic field produced by the magnet. As force (**F**, Fxyz) is applied to the upper surface, the elastomer layer deforms, allowing displacement of the magnet (**D**, Dxyz). This causes variations in the magnetic field (**B**, Bxyz) at the position of the sensor. Individual nodes were placed with 15 mm separation to reduce magnetic crosstalk between adjacent nodes. At 15 mm separation, magnetic crosstalk between nodes was reduced below 0.1% at full scale loading in all axes and was treated as negligible.

All sensors were calibrated using a single representative dataset, with the resultant calibration applied to each sensing node in the sensing array. A two‐step calibration process was used to negate errors caused by manufacturing inconsistencies (e.g., inter‐node variation in magnet positioning). First, the magnetic field at the sensor was calibrated to detect the magnet's position (Equation 1), with the initial position being tared (Equation 2). Then, the magnets' relative movements were calibrated to the force applied to the surface of the elastomer (Equation 3). Neural network fitting was used to generate calibration functions, owing to its effectiveness in fitting highly non‐linear relationships observed in similar sensing systems [17].

(1) D=f1(B)

(2) D_t=D–D_0

(3) F=f2(D_t)

To generate the training dataset for the position calibration, a magnet was attached to a three‐axis linear rail. The magnet was centralised over the Hall‐effect sensor and then swept in a 3D space above. The total space equalled 1 × 1 × 4 mm. To generate training data for the applied force, a smaller, 3 × 3 node version of the elastomer was produced using the above method and mounted into a 3‐axis loading rig (Figure 1D). The system swept through several different loads in each axis to generate training data (Up to 255 kPa normal, 120 kPa shear). The calibration setup used an identical configuration to each node of the platform, including the same batch of elastomer and magnet (to mitigate against manufacturing variability). A verification pass was conducted using the calibration system based on our prior sensor development, demonstrating a hysteresis error of 5% in each axis and 1% variability between repeated load cycles [17].

#### 2.1.2 Elastomer Fabrication and Mechanics

To ensure consistency across the surface of the footplate, a method was devised to embed the 128 magnets within a single sheet of elastomer (Renishaw 8891 flexible casting resin, mixed at A100:B100:C300, UV stable, 10 MPa tensile strength, medically approved). To do this, a two‐stage moulding process was devised, utilising a vacuum caster (5/01 ULC vacuum casting machine], Renishaw). The first stage casts a layer of elastomer with an array of negative features on the surface. The magnets were then inserted into each space, before a second layer of elastomer was cast above them, to fully encapsulate them (Figure 2).

**FIGURE 2:**
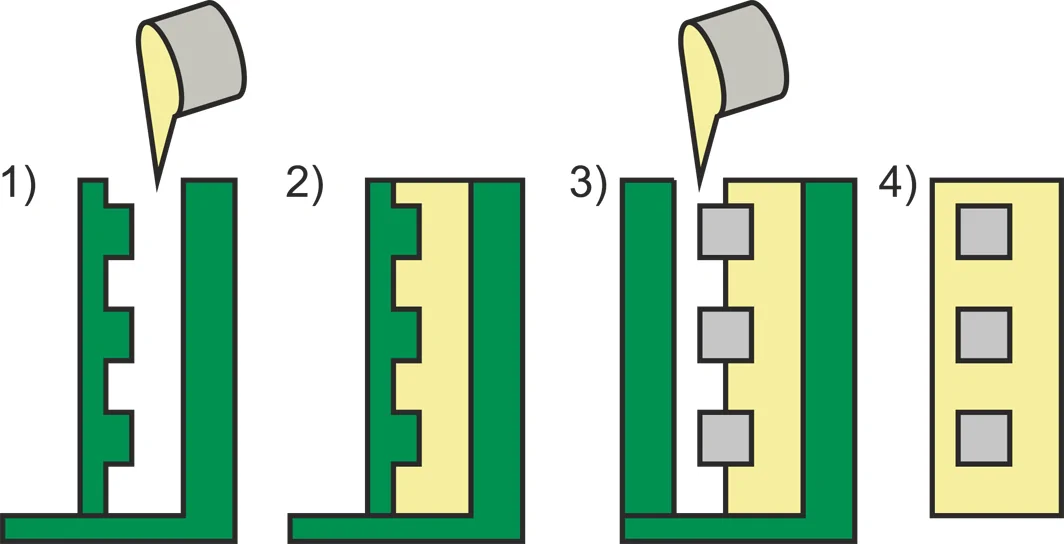
Manufacturing process for the elastomer sheet. (1) A 2‐piece mould to produce negatives, (2) the positive mould face is removed, (3) magnets inserted into negative spaces, and remaining elastomer poured to produce (4), the final structure.

#### 2.1.3 Platform Structure

A metal platform was designed to both mount the components together and to support the load of the participant. Within the platform, an array of pillars was added creating space for the sensing PCB to be mounted in a manner that fully isolated it from any stresses caused by the loading of the plate. The assembled plate was sunk into the central tile of a walkway consisting of EVA foam tiles, allowing participants to walk across a level walkway, thus measuring shear stresses during normal gait.

### 2.2 Electronics and Data Processing

To enable the recording of the magnetic field data, two custom PCBs were produced. First, a board with 128 Hall Effect sensors was produced and arranged in an 8 × 16 grid to produce an active sensing area of 120 × 240 mm (with 5 mm spacing between sensors). Each sensor was spaced to sit directly below the associated magnet, fitting in to spaces within the top plate of the platform. To allow for 100 Hz data recording, the sensors were arranged across four ‘banks’. Each bank was connected to a different communication channel (an i2 c line) on the microcontroller, allowing for asynchronous measurement of the chips. Each bank consisted of a 4‐way multiplexer, with each branch having 8 individually addressed chips (Figure 3).

**FIGURE 3:**
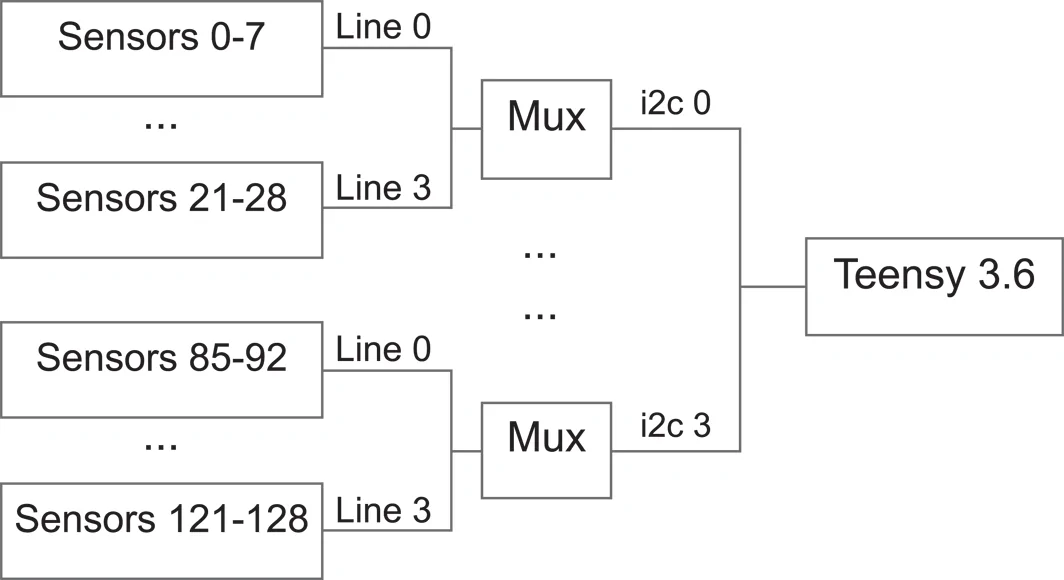
Electronic control diagram. A microcontroller (Teensy 3.6) has four i2 c busses, each connecting to a four‐way multiplexer. Each multiplexer output is connected to a bank of 8 sensors, totalling 128.

A second PCB was designed to power and control the system. A microcontroller (Teensy 3.6, PJRC) collated the data from the 128 nodes, recording to a buffer in an onboard SD card. Once a measurement was complete, the data was streamed to a connected PC running a custom software for data collection and processing. Data were recorded by a custom LabVIEW script to pull the sensor data and save to file.

To process the raw data, a custom LabVIEW script was developed to detect footsteps and identify individual data metrics. First, the timeseries data was averaged across the full plate to identify the timeframe of peak forefoot contact. Then, the calibrated force and shear output was calculated for each node, leaving a grid of data. A clinical team member then manually aligned a mask over the area loaded by the foot to segment the data to specific zones of the foot. To aid in visualisation, the data were B‐spline interpolated to a spacing of 1 mm and presented as an image for masking to take place. Finally, the peak and mean loads were calculated for each anatomical area.

#### 2.2.1 Clinical Studies

Patients were recruited from routine outpatient rheumatology clinics at Chapel Allerton Hospital (CAH) in Leeds and tested in the adjacent NIHR Leeds BRC gait laboratory. All patients and normal control participants gave their signed consent to take part in the study. Ethical approval was obtained for this study (London‐Bromley REC, reference 19/LO/0944).

Patients completed several questionnaires which are in standard use in PsA clinics. These included the Health Assessment Questionnaire (HAQ), the Psoriatic arthritis Quality of Life tool (PsAQoL), and the Psoriatic arthritis Impact of Disease tool (PSAID12) in addition to providing a global measure of their disease status. Demographic data were collected in addition to other clinical data including the 66 swollen and 68 tender joint count, the Leeds Dactylitis Index [18], the Leeds Enthesitis index [19], the Foot Posture Index [20], an assessment of shoe fit, and a physician global assessment of their disease. Patients were excluded if they had undergone foot and ankle surgery, if they were unable to walk 20 m unaided, or if they had significant co‐morbidity likely to affect the foot, such as diabetes, neurological disease, or peripheral vascular disease.

Participants walked barefoot over a 10‐m walkway at the participant's preferred speed and cadence [21]. A common two‐step protocol was used for the collection of barefoot plantar pressure data, and three representative steps were collected for each foot accordingly. A clear footstep was determined by the clinicianwho were experienced research podiatrists (RAW, JD). A trial was included if the participants walked at their normal walking speed, and both the metatarsal phalangeal joints and toes made a clean contact with the ESCAPES platform (Figure 1B). All steps fouling the margins of the pressure plate were excluded. Peak pressure (KpA) and shear force (*x*, *y* and *z* axis) were analysed at the hallux, all the lesser toes and the foot region as a whole [15]. To capture the interaction between the toe and metatarsal, a mask was used to capture both structures as individual rays [1, 2, 3, 4, 5]. Masking was conducted by two trained clinical researchers with experience in masking foot plantar pressure data (Wilkins et al., [11]), guided with reference to a video of the foot strike on the sensor platform, as shown in Figure 4. Participant data were divided between the researchers; for each sample, one researcher added the mask and the second checked and verified the outcome. Data were reported as a mean of the three trials and normalised to the participant's body weight [15, 22].

**FIGURE 4:**
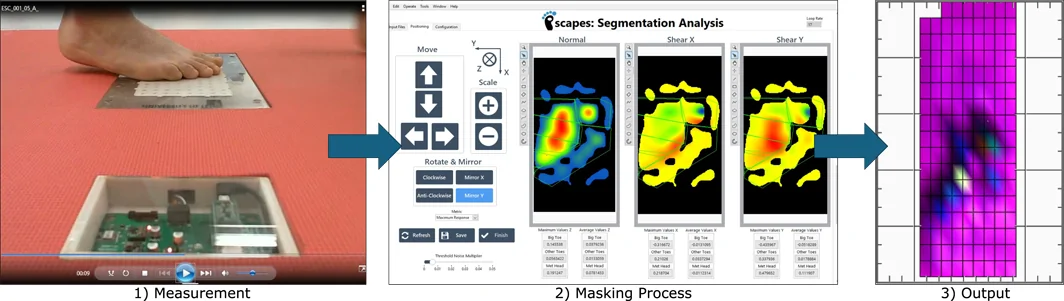
Masking and Data Analysis process used in ESCAPES.

As this was a pilot feasibility research study, no formal sample size calculations were made. We aimed to collect 20 participants from each of the following groups: normal controls, active (current) dactylitis of the toes, a history of dactylitis of the toes, and people with PsA without any current or historical dactylitis.

#### 2.2.2 Statistics

This was a pilot study and therefore not powered to examine statistical significance. Nevertheless, groups were compared using one way analysis of variance, with post hoc tests examining differences between each group. The large number of observations necessitated a correction for multiple comparisons and a ‘corrected’ significance level of 0.002 was used.

## 3 Results

The study was under‐recruited due to the Covid‐19 pandemic. Table 1 details the clinical and demographic details of participants recruited. Patients with PsA and no history of dactylitis were older, had a longer duration of disease and had less active disease than the other groups. Patients with current dactylitis had more active disease (as measured by the tender and swollen joint counts, and the global disease activity scores) than the other groups. Generally, there were no differences between the groups for the FPI and the shoe fit assessment (Table 1).

**TABLE 1:** Demographic and clinical features of the participants.

Variable	Normal controls *n* = 12	Active (current) dactylitis *n* = 6	Past history of dactylitis *n* = 17	PsA with no history of dactylitis *n* = 12
Age (y)	38.5 (14.0)	44.0 (13.3)	42.9 (14.6)	56.4 (13.2)
Sex	M 8/F 4	M 5/F 1	M 9/F 8	M 7/F 5
Duration of PsA (y)	n/a	5.0 (4.3)	2.4 (3.5)	16.6 (12.7)
Ethnicity: White north european	12 (100)	6 (100)	16 (94)	12 (100)
South asian	0	0	1 (6)	0
BMI	23.9 (3.8)	26.1 (4.6)	27.1 (5.2)	32.1 (5.5)
Clinical variables				
Tender joint count (0–68)	n/a	8.0 (8.2)	7.4 (10.2)	4.4 (8.2)
Swollen joint count (0–66)	n/a	5.2 (3.9)	2.1 (3.0)	1.2 (2.2)
LDI	n/a	116.6 (11.9)	n/a	n/a
LEI (0–6)	n/a	0.75 (0.96)	1.0 (0.89)	2.0 (0)
Physician global score (0–10 with 10 being worst)	n/a	4.6 (2.8)	2.9 (2.5)	3.1 (1.8)
Shoe fit: Good	9 (75)	3 (50)	15 (88)	7 (58)
Too short	3 (25)	1 (17)	2 (12)	4 (33)
Too long	0	2 (33)	0	1 (8)
FPI (−4 to +4): Left foot	2.2 (2.4)	3.5 (2.4)	2.4 (2.0)	1.8 (3.5)
Right foot	2.4 (2.6)	4.2 (3.7)	2.7 (2.7)	1.5 (3.7)
CRP (mg/dL)	n/a	6.3 (5.1)	2.1 (2.9)	9.9 (15.9)
Medication: NSAIDs	n/a	3 (50)	5 (30)	3 (25)
csDMARDs	n/a	1 (17)	5 (30)	7 (58)
bDMARDs	n/a	2 (33)	10 (59)	6 (50)
Oral steroids	n/a	0	1 (6)	0
Patient reported outcomes				
PsAQoL (0–22)	n/a	4.7 (5.1)	6.0 (5.8)	6.9 (5.6)
PSAID (0–10)	n/a	3.5 (2.3)	3.6 (2.8)	3.6 (2.2)
HAQ (0–3)	n/a	0.44 (0.65)	0.40 (0.52)	0.63 (0.68)
Patient global assessment (0–10)	n/a	4.3 (2.3)	3.5 (2.8)	3.5 (1.9)

*Note:* Figures are mean (sd) or *N*.

Abbreviations: bDMARD, biologic disease modifying drug; BMI, body mass index kg/m^2^; cdDMARD, conventional synthetic disease modifying drug; CRP, C reactive protein; FPI, Foot posture index; HAQ, health assessment indexLDI, Leeds Dactylitis Index; LEI, Leeds Enthesitis Index; NSAID, non‐steroidal anti‐inflammatory drug; PSAID, psoriatic arthritis impact of disease; PsAQoL, psoriatic arthritis quality of life.

Tables 2 and 3 show the mean maximum stresses in each axis, in each region, for each foot, by diagnostic group. The indicated stresses were also compared individually for the digit actively inflamed, but no difference between the individual stresses and the mean for that digit in the other groups was found (data not shown). No consistent differences between the diagnostic groups were observed. In the right foot, stresses were generally higher in the control group in all axes, though the opposite was found in the left foot. None of the post hoc tests between individual groups were significant. As to the individual rays, in the left foot shear stresses were higher for rays 2–4 compared to normal subjects, though this was not the case for the right foot.

**TABLE 2:** Right foot: mean maximum shear stress and pressure (+/− sd) in kPa normalised to 70 kg individual weight.

Region	Normal controls *n* = 12	Active (current) dactylitis *n* = 6	Past history of dactylitis *n* = 17	PsA with no history of dactylitis *n* = 12	F	*p*
Hallux: *x* Axis	−38.3 ± 43.0	−27.1 ± 43.7	4.6 ± 55.8	9.9 ± 22.0	3.0	ns
*y* axis	−42.8 ± 25.4	−30.0 ± 17.7	−33.3 ± 19.9	−19.7 ± 17.1	2.5	ns
*Z* axis	177.4 ± 167.4	104.4 ± 65.0	158.9 ± 108.6	129.6 ± 74.2	0.7	ns
Toes: *x* Axis	−63.4 ± 56.9	−50.9 ± 45.3	5.8 ± 51.6	−23.4 ± 54.4	3.9	ns
*y* axis	−67.3 ± 47.9	−40.1 ± 17.3	−28.6 ± 26.4	−33.1 ± 23.2	3.4	ns
*Z* axis	200.4 ± 174.8	135.4 ± 77.2	158.3 ± 74.4	155.2 ± 53.0	0.6	ns
Foot: *x* Axis	68.8 ± 78.3	84.4 ± 51.1	34.6 ± 49.5	33.9 ± 50.3	1.7	ns
*y* axis	−94.1 ± 48.3	−54.2 ± 17.4	−36.2 ± 24.5	−40.4 ± 25.6	7.9	< 0.001
Z axis	473.1 ± 222.7	367.1 ± 171.2	309.3 ± 105.6	279.4 ± 91.7	3.7	ns
1st ray: *x* axis	39.3 ± 48.3	9.7 ± 24.0	6.5 ± 60.9	30.2 ± 33.3	1.2	ns
*y* axis	−40.3 ± 35.3	−34.8 ± 21.4	−34.2 ± 18.7	−26.5 ± 21.8	0.6	ns
Z axis	338.9 ± 188.2	193.8 ± 90.1	211.0 ± 104.1	172.7 ± 66.3	4.0	0.015
2nd ray: *x* axis	72.3 ± 79.9	33.9 ± 54.4	18.9 ± 54.7	43.1 ± 50.7	1.6	ns
*y* axis	−70.4 ± 36.9	−44.2 ± 20.1	−31.1 ± 23.1	−27.3 ± 18.1	6.4	0.001
*Z* axis	413.6 ± 191.1	292.2 ± 111.6	247.5 ± 102.3	243.1 ± 86.8	4.3	ns
3rd ray: *x* axis	27.9 ± 87.7	49.6 ± 50.5	36.5 ± 53.1	19.1 ± 45.8	0.4	ns
*y* axis	−80.3 ± 33.7	−47.4 ± 21.9	−31.6 ± 26.5	−30.4 ± 24.4	8.0	< 0.001
*Z* axis	380.2 ± 231.5	293.7 ± 176.0	238.0 ± 84.7	252.0 ± 88.5	2.2	ns
4th ray: *x* axis	−67.7 ± 62.4	43.5 ± 46.9	21.5 ± 45.7	−8.2 ± 50.7	8.0	< 0.001
*y* axis	−74.2 ± 51.9	−43.3 ± 19.8	−30.3 ± 22.8	−9.9 ± 7.3	4.2	ns
*Z* axis	255.0 ± 247.7	243.6 ± 180.1	183.9 ± 69.8	172.6 ± 54.7	0.8	ns
5th ray: *x* axis	−59.2 ± 53.8	−30.9 ± 42.9	4.1 ± 43.9	−22.5 ± 48.3	3.6	ns
*y* axis	−54.9 ± 45.8	−31.8 ± 11.9	−22.8 ± 16.5	−27.7 ± 25.5	2.9	ns
*Z* axis	164.3 ± 179.1	143.2 ± 96.2	125.6 ± 55.6	123.9 ± 53.1	0.4	ns

*Note: X* axis: mediolateral shear stress (positive is medial); *Y* axis: anterior posterior shear stress (positive is anterior); *Z* axis: perpendicular stress (pressure). *p* values are actual (corrected significance level 0.002).

**TABLE 3:** Left foot: mean maximum shear stress and pressure (+/− sd) in KpA normalised to 70 kg individual weight.

Region	Normal controls *n* = 12	Active (current) dactylitis *n* = 6	Past history of dactylitis *n* = 17	PsA with no history of dactylitis *n* = 12	F	*p*
Hallux: *x* Axis	−11.3 ± 46.0	−31.9 ± 48.3	−11.9 ± 33.6	−1.5 ± 17.5	0.9	ns
*y* axis	−21.4 ± 19.6	−45.4 ± 28.9	−33.3 ± 13.6	−21.8 ± 15.3	3.1	ns
*Z* axis	102.1 ± 113.3	181.2 ± 155.6	124.2 ± 68.2	87.2 ± 83.1	1.3	ns
Toes: *x* Axis	−20.2 ± 47.6	−26.9 ± 22.5	8.2 ± 42.3	5.7 ± 53.3	1.4	ns
*y* axis	−22.7 ± 17.3	−47.2 ± 42.0	−23.1 ± 16.8	−19.1 ± 23.0	2.1	ns
*Z* axis	91.1 ± 92.6	173.5 ± 164.6	167.8 ± 107.8	151.9 ± 119.2	1.0	ns
Foot: *x* Axis	−3.8 ± 50.0	15.9 ± 67.9	47.7 ± 74.9	12.8 ± 83.1	1.2	ns
*y* axis	−24.6 ± 20.6	−55.8 ± 56.6	−63.2 ± 17.8	−54.7 ± 17.5	4.7	ns
*Z* axis	123.9 ± 102.4	293.4 ± 275.1	334.1 ± 140.9	239.3 ± 137.8	3.8	ns
1st ray: *x* axis	16.3 ± 47.4	−16.2 ± 50.4	0.1 ± 54.8	−8.7 ± 43.2	0.7	ns
*y* axis	−23.8 ± 23.8	−41.3 ± 28.1	−49.7 ± 24.7	−39.8 ± 24.3	2.2	ns
*Z* axis	129.8 ± 116.4	247.1 ± 222.4	208.5 ± 79.4	137.9 ± 86.2	2.1	ns
2nd ray: *x* axis	−14.8 ± 47.9	−31.7 ± 36.2	15.1 ± 55.6	14.1 ± 57.2	2.3	ns
*y* axis	−22.8 ± 18.8	−50.8 ± 45.1	−56.0 ± 14.7	−44.6 ± 24.8	3.9	ns
*Z* axis	88.0 ± 67.6	258.0 ± 276.5	247.9 ± 97.2	199.5 ± 122.1	3.3	ns
3rd ray: *x* axis	−1.7 ± 44.0	17.0 ± 84.0	32.6 ± 63.1	23.9 ± 61.1	0.6	ns
*y* axis	−15.9 ± 13.9	−42.5 ± 47.8	−32.3 ± 23.4	−34.8 ± 14.1	1.9	ns
*Z* axis	88.8 ± 94.0	235.2 ± 288.2	274.1 ± 153.4	209.7 ± 136.5	2.7	ns
4th ray: *x* axis	−16.6 ± 36.7	−35.2 ± 23.9	−28.3 ± 12.4	−27.4 ± 17.7	0.4	ns
*y* axis	−16.2 ± 13.0	−35.2 ± 23.9	−28.3 ± 12.4	−27.4 ± 17.7	2.0	ns
*Z* axis	64.8 ± 56.9	168.6 ± 168.0	186.0 ± 132.1	157.5 ± 117.5	2.2	ns
5th ray: *x* axis	−28.6 ± 26.4	−22.9 ± 24.5	2.4 ± 42.9	−13.3 ± 49.3	1.4	ns
*y* axis	−19.8 ± 14.2	−25.1 ± 12.1	−24.1 ± 9.5	−27.2 ± 13.5	0.7	ns
*Z* axis	60.1 ± 42.7	84.5 ± 18.1	137.8 ± 103.4	123.7 ± 88.8	2.1	ns

*Note: X* axis: mediolateral shear stress (positive is medial); *Y* axis: anterior posterior shear stress (positive is anterior); *Z* axis: perpendicular stress (pressure). None of the comparisons were significant.

## 4 Discussion

In this study, a novel platform for measuring stresses in the foot was developed and, in an associated clinical application in patients with PsA, no consistent differences in stresses were found between control patients and disease groups. The latter result was not surprising given the small size of the groups studied. From the perspective of the trauma hypothesis, it is worth noting that the shear stresses in the 2nd to 4th toe ‘rays’ were higher than the hallux or the 5th ray, at least in the left foot. In future studies, with more participants, it might also be worth looking at a combination of pressure and shear to investigate abnormalities in this population, as well as employing other ways of analysing the data, such as data reduction or artificial intelligence.

Dactylitis is a complex condition in which inflammation is found in most of the tissues of the digit. It has been hypothesised that local trauma may be a trigger for this pathology in this disease population and, given the predilection for the 2nd and 4th toes, the trauma may be more prominent in these digits. In this small pilot study, shear stresses were higher in the second to fourth digits for the left foot, which supports this hypothesis, though larger studies are needed. One problem when studying in this area is that although higher shear stresses may be an initiating factor, once inflammation in the digit has developed, it is likely that the patient will tend to ‘favour’ that digit/foot when walking to reduce the stresses on that painful digit. Such a phenomenon will thus confound studies of this kind.

A profound asymmetry in stresses between the right and left feet was observed in this study, with overall larger stresses in the right foot. This difference applied to stresses in all three axes, and between normal and disease groups. Most people are right foot dominant so that walking to place the foot on a small platform is easier if it is the dominant foot that is placed therein. It is possible that the difference in stresses results from a more hesitant placement of the non‐dominant foot, but more work is needed to confirm this. A previous study found asymmetry of pressure profiles between the feet of ‘normal’ people but did not relate this to foot dominance [23].

The novel device developed for this study brings several key advantages. Firstly, it provides multi‐axis measurements of plantar loading from point contact, allowing detailed assessments of both pressure and shear forces. This capability addresses significant limitations of existing devices that predominantly measure plantar pressures alone. In the data processing, manual masking of the plantar data gives a good estimation of foot location but can introduce variability and potential subjectivity. Other devices for plantar pressure alone, such as ‘emed’ (https://novel.de/products/emed/), have automated this step although further data are required to do this.

However, there are some disadvantages to consider. The relatively large size and weight of the sensing platform limits its portability, potentially restricting its use primarily to specialised gait laboratories rather than general clinical practice. Although larger than previous devices, with sufficient area to measure a full foot, in practice this was hard to achieve, particularly with participants suffering from pain. Timing the gait to hit the plate perfectly was difficult; therefore, we opted to rotate at 90° to measure only the forefoot to limit the number of repeats the patient was asked to perform. However, the system is designed to be modular so that future iterations could combine four sensing plates to increase the measurement area and ease the challenge hitting the sensing area. Similarly, increasing measurement resolution (the density of sensing nodes) will focus on advances in the underlying Hall‐effect sensors, enabling more spatially precise measurements of plantar load. Whilst participants were encouraged to rest between trials using a perching stool, the small sensor area of the shear plate may have had an impact on the number of trails undertaken; this may have resulted in a higher number of trials and increased participant fatigue. Increasing the platform size may decrease the overall number of trials, and the potential for participant fatigue.

## 5 Conclusion

In conclusion, the observation of higher shear stresses in the second to fourth toes aligns with the hypothesis that repetitive local trauma contributes to the onset of inflammation in these digits. Nonetheless, interpreting shear data is complicated by alterations in gait patterns associated with pain, as individuals may subconsciously modify their walking mechanics to reduce discomfort. Such altered gait patterns pose a significant challenge for the interpretation of biomechanical data, potentially masking or exaggerating the true mechanical stresses experienced during typical ambulation. Future studies should consider methods to objectively quantify gait adaptations or employ longitudinal designs to differentiate between cause and effect in plantar loading variations.

## Author Contributions

**Dominic Jones:** methodology, software, funding acquisition, writing – original draft, writing – review and editing. **Lefan Wang:** methodology, software, writing – review and editing. **Max Houghton:** methodology, writing – original draft, writing – review and editing. **Jennifer Jones:** funding acquisition, data curation, writing – review and editing. **Richard A Wilkins:** funding acquisition, data curation, writing – review and editing. **Heidi J Siddle:** supervision, writing – original draft, writing – review and editing. **Graham J. Chapman:** conceptualization, funding acquisition, methodology, data acquisition, writing – review and editing. **Ali Alazmani:** methodology, software, writing – original draft, writing – review and editing. **Peter Culmer:** methodology, supervision, funding acquisition, writing – original draft, writing – review and editing. **Philip S Helliwell:** conceptualization, funding acquisition, data curation, analysis, writing – original draft, writing – review and editing.

## Funding

The development of the platform, and the clinical studies, were funded by a pilot research grant from the Group for Research and Assessment of Psoriasis and Psoriatic arthritis (GRAPPA) and carried out at the National Institute for Health and Care Research (NIHR) Leeds Biomedical Research Centre (BRC) (NIHR203331). The views expressed are those of the author(s) and not necessarily those of the GRAPPA, the NIHR, or the Department of Health and Social Care.

## Conflicts of Interest

The authors declare no conflicts of interest.

## Data Availability

The data that support the findings of this study are available from the corresponding author upon reasonable request.
